# Clinical and functional status of patients with severe COVID-19 pneumonia: an observational study at 2–3 months following discharge

**DOI:** 10.3389/fresc.2023.1248869

**Published:** 2023-08-21

**Authors:** Inmaculada Castillo Sánchez, Julia Tárrega Camarasa, Enric Barbeta Sánchez, Vinicius Rosa Oliveira

**Affiliations:** ^1^ Pneumology Department, Granollers General Hospital, Barcelona, Spain; ^2^ Faculty of Health Sciences and Welfare, Universitat de Vic–Universitat Central de Catalunya (UVic-UCC), Vic, Spain; ^3^ Faculty of Medicine and Health Sciences, Universitat Internacional de Catalunya, Sant Cugat del Vallès, Spain; ^4^ Research Group on Methodology, Methods, Models and Outcomes of Health and Social Sciences (M_3_O), UVic-UCC, Vic, Spain

**Keywords:** COVID-19, diaphragm ultrasound, follow-up, functional assessment, lung ultrasound, mechanical ventilation

## Abstract

**Introduction:**

Critically ill COVID-19 patients present long-term sequelae that affect their everyday life. This study aimed to describe the clinical and functional status of patients with severe COVID-19 pneumonia at 2–3 months post discharge from a Spanish critical care unit.

**Methods:**

We collected retrospective data from 58 patients admitted to the critical care unit with diagnosis of severe respiratory failure due to COVID-19. Only patients who required invasive (IMV) or noninvasive ventilation (NIV) during their hospital stay were included. The following data were collected 2–3 months after hospital discharge: respiratory signs and symptoms, lung ultrasound (LUS) and diaphragm ultrasound images, blood test analysis, lung function parameters (spirometry and DLCO), exercise capacity (6 min walk test and sit-to-stand test), level of physical activity and health-related quality of life.

**Results:**

We found clinical symptoms and lung structural alterations in LUS images of 26 patients (48.1%). Those presenting LUS abnormalities had longer length of stay in hospital (*p *= 0.026), functional alterations in spirometry (*p *< 0.01) and decreased diaphragm excursion (*p *= 0.029). No significant alterations were observed in blood test analysis, exercise capacity, level of physical activity and health-related quality of life.

**Conclusions:**

A significant part of the patients admitted to a critical care unit continue to present clinical symptoms, pulmonary morphological abnormalities, and lung function alterations 2–3 months post discharge. This study corroborates that assessing the functional status of the survivors is essential to monitor the evolution of pulmonary sequelae.

## 1. Introduction

COVID-19 can cause severe respiratory failure and manifest as acute respiratory distress syndrome (ARDS) (1). Mechanical ventilation (IMV) is the treatment of choice in most severe cases of ARDS, and those who receive IMV are at increased risk of mortality (2, 3). Notwithstanding, the great magnitude of the first wave of the pandemic encouraged the creation of intermediate respiratory care units (IRCU) boosting the use of non-invasive ventilation (NIV) to attend the high demand of the health system (4, 5).

Lung ultrasound (LUS) application has been clearly intensified during COVID-19 pandemic as a powerful noninvasive bedside tool that allows a quick and safe assessment to diagnose interstitial-alveolar syndrome, lung consolidation or pleural effusion (6, 7). Likewise, application of diaphragm ultrasound gained importance to detect structural deteriorations due to prolonged IMV and also due to pathophysiological mechanisms observed in COVID-19 (8–10). In patients in the critical care unit, diaphragm dysfunction due to COVID-19 was estimated in 10% (9). Despite the use of diaphragm and LUS is widely accepted in the acute phase of COVID-19 (8), its utility still needs to be defined in the long-term follow-up.

In COVID-19 survivors, the persistence of symptoms still places a burden on the society. Even individuals infected with mild COVID-19 depict prolonged symptoms that affect basic and instrumental activities of daily living (11–13). Approximately half of patients recovering from COVID-19 report chronic dyspnea 2–3 months after infection (14, 15). On top of that, presence of chronic symptoms reduces the quality of life and work productivity in survivors (14, 16).

To date, some studies have reported the long-term outcomes on physical fitness of patients severely affected by COVID-19 (17–20). However, the available evidence is conflicting on the fully fitness recovery of these patients after hospital discharge. Furthermore, the relationship between lung morphological alterations and functional findings after discharge of critically ill patients with COVID-19 has not been yet elucidated thoroughly. We hypothesized that lung morphological alterations caused by severe COVID-19 pneumonia could be detected 2–3 months after discharge and related to changes in lung function and physical fitness. The objective of this study was to describe the clinical and functional status of patients with severe COVID-19 pneumonia at 2–3 months post discharge from a Spanish critical care unit.

## 2. Materials and methods

In this single-center retrospective descriptive study, the medical records of patients admitted to the intensive care unit (ICU) or the intermediate respiratory care unit (IRCU) of Granollers General Hospital (Spain) were analyzed. The study protocol was approved by the Research Ethics Committee of Granollers General Hospital with an exemption for informed consent. The study was conducted in accordance with The Code of Ethics of the World Medical Association (Declaration of Helsinki) and the Strengthening the Reporting of Observational Studies in Epidemiology (STROBE) statement (21).

We included patients admitted to the ICU or IRCU from March to May 2020 due to COVID-19 associated severe respiratory failure who required use of IMV or NIV. The exclusion criteria were patients treated with oxygen therapy only, those who presented any contraindication to perform the follow-up functional tests, and those who did not attend the follow-up visit 2–3 months after discharge.

### 2.1. Procedure

Two to three months after hospital discharge, an outpatient follow-up visit was conducted at the Pneumology Department of Granollers General Hospital by a pulmonologist and a physiotherapist to assess the clinical and functional status of the participants. Data were collected in a Microsoft Excel Spreadsheet (.xlsx) respecting the anonymity of the participants.

The clinical history of each participant was reviewed to obtain gender (male/female), age (years), body mass index (BMI, kg/m^2^), comorbidities, respiratory signs and symptoms, duration of IMV or NIV, tracheostomy (yes/no), and length of stay in the ICU or IRCU. The participants were requested to report any sign or symptom of dyspnea, mucus hypersecretion or cough episodes.

Blood test analysis was used to study the inflammatory and coagulative parameters D-dimer, C-reactive protein, PT, and tPA.

### 2.2. Lung ultrasound

Lung ultrasound exam was performed with the equipment TOSHIBA Xario 200 (Tokyo, Japan), using a convex probe of frequency ranging from 2 to 6 Hz. It was measured at 6 points on each hemithorax (posteriorly, laterally, and anteriorly) with the patient seated (22). The collection of ultrasound findings was determined by using a score from 0 to 3 validated by patients with COVID-19, as follows: 0) A lines or <3 isolated B lines; (1) at least 3 B lines or confluent B-lines occupy <50% screen; *2) confluent B lines >50% of the screen with or without irregular pleura; and (3) consolidation (22). These categories were further recoded into 2 groups: normal (score 0) and abnormal (scores 1, 2 and 3).

### 2.3. Diaphragm ultrasound

Diaphragm ultrasound exam was performed with the same equipment as used for lung ultrasound, using a convex probe of frequency ranging from 2 to 6 MHz. The patient was assessed in supine position at 45° using the clavicular midline subcostal approach. By using the M mode, the diaphragm excursion was measured in centimeters during shallow breathing, deep breathing, and sniff maneuver. The diaphragm thickness was measured in millimeters with a high-frequency linear probe of 6–13 MHz, at the anterior axillary line between 8th-10th intercostal spaces. In B mode, 3 measurements were made at the end of a calm expiration using reference values (10, 23).

### 2.4. Lung function tests

Lung function tests were performed with Vyntus PC spirometer (MasterLab, Jaeger, Würzburg, Germany) in basal conditions and after the administration of 200 micrograms of salbutamol. We recorded the forced vital capacity (FVC, in ml), forced expiratory volume in the first second (FEV1, in mL) and FEV1/FVC ratio, in %. The reference values were based on the study by (24) in the Mediterranean population.

The diffusing capacity of the lungs for carbon monoxide (DLCO) and the carbon monoxide transfer coefficient (KCO) were measured using the single breathing method with the gas meter integrated in the Masterlab DLCO equipment (Masterlab, Jaeger, Würzburg, Alemania). We used the reference values described for the Mediterranean population (25).

### 2.5. 6MWT

Exercise capacity was assessed through the 6-minute walk test (6MWT), conducted by a trained nurse. Patients had to walk on a 30 m-long aisle at a pace similar to the activities of daily living, with a pulse oximeter placed in the finger to measure the heart rate and oxygen saturation (26). The walked distance was recorded in meters, and the percent predicted values were calculated using reference equations for the Spanish population (27). We collected the heart rate (HR) at baseline, the highest HR achieved in the 6MWT, the lowest oxygen saturation (SpO_2_), and the difference between the baseline SpO_2_ value and the lowest SpO_2_ value during the 6MWT (6MWT desaturation %).

### 2.6. Sit-to-stand test

The 30 s sit-to-stand (30 s-STS) test was used to assess submaximal exercise capacity. It was conducted using a 43 cm-high chair without armrests. The participants were instructed to start the test seated on a 43 cm-high chair with both feet on the floor at an angle slightly back from the knees and the arms crossed in front of the chest. Then, they were asked to stand up, as many times as they could in 30 s. The number of times they stood up was recorded (28).

### 2.7. Self-administered international physical activity questionnaire (IPAQ)

The short version of this questionnaire (IPAQ-SF) was validated by (29). The questions refer to the “last 7 days”, consisting of 9 items and provides information on the time spent on walking, activities of moderate and vigorous intensity, and sedentary activities. It allows to record the values in total time and caloric consumption. There are 3 specific characteristics of activity: intensity (mild, moderate or vigorous), frequency (measured in days per week) and duration (time per day). The short version assesses walking, moderate and intense activities. The final result is classified into 3 categories: Intense category: Intense activity minimum 3 days/week reaching a minimum of total physical activity of 1,500 METs-min/week, or 7 days of combining walking, moderate or intense activity reaching a minimum of total physical activity of 3,000 METs-min/week. Moderate category: ≥3 days of intense activity of at least 20 min/day, or ≥5 days of moderate activity and/or walking of at least 30 min/day, or ≥5 days of any combination of walking. Mod. or intense that reaches a total physical activity of minimum 600 METs-min/week. Low category: All subjects who do not meet the inclusion criteria of the previous categories are included (30).

### 2.8. Health-related quality of life

Health-related quality of life was assessed through the abbreviated King Interstitial Lung Disease Questionnaire (K-BILD), consisting of 15 questions with 7 answer options covering 3 domains: psychological, shortness of breath, activities, and thoracic symptoms. The total score is 100, with higher scores meaning best health status (31).

### 2.9. Statistical analysis

Data obtained during the study were coded at the end of the collection, and then analyzed using the Statistical Analysis System (SAS) software for Windows, version 9.4 (SAS Institute, Cary, SC, USA). Descriptive analysis was carried out indicating absolute and relative frequencies for categorical variables and mean and standard deviation (SD) for quantitative variables. Comparisons between normal and abnormal lung ultrasound groups in relation to functional parameters were assessed with Student's *t*-test. A *post hoc* power analysis using observed estimate of effect size (Cohen's d) was conducted to determine the statistical power of the study. *p *< 0.05 was considered statistically significant.

## 3. Results

The total sample consisted of 58 patients with a mean age of 62.2 (SD 10.2) years, mostly male (67.2%) with the following signs and symptoms at 2–3 months of follow-up: mucus hypersecretion (31.0%) dyspnea (29.3%), and dry cough (20.7%). The average length of stay in the critical unit was 29.9 days (SD 20.3). Participants with abnormal findings in LUS stayed longer in the UCI or IRCU when compared to those with normal LUS findings (*p *= 0.026). The most frequent comorbidities were hypertension (46.6%), diabetes (29.3%), and obesity (17.2%), as described in Table 1.

**Table 1:** Sample characteristics.

Male gender, *n* (%)	**39** **(67.2%)**
Age (years), mean (SD)	**62.2** **(10.2)**
BMI (kg/m^2^), mean (SD)	**29.8** **(5.1)**
Mucus hypersecretion, *n* (%)	**18** **(31.0%)**
Dyspnea, *n* (%)	**17** **(29.3%)**
Dry cough, *n* (%)	**12** **(20.7%)**
Invasive mechanical ventilation, *n* (%)	**37** **(63.8%)**
Noninvasive ventilation, *n* (%)	**29** **(50.0%)**
Traqueostomy, *n* (%)	**17** **(29.3%)**
Duration of IMV/NIV (days), mean (SD)	**16.8** **(13.9)**
ICU or IRCU length of stay (days), mean (SD)	**29.9** **(20.3)**
Comorbidities, *n* (%)	
Hypertension	**27** **(46.6)**
Diabetes	**17** **(29.3)**
Obesity	**10** **(17.2)**
Congestive heart failure	**5** **(8.6)**
Obstructive sleep apnea syndrome	**5** **(8.6)**
Chronic obstructive pulmonary disease	**3** **(5.2)**
Other	**19** **(32.8)**

Sample characteristics of 58 patients with severe COVID-19 pneumonia 2–3 months following discharge. BMI, body mass index; ICU, intensive care unit; IMV, invasive mechanical ventilation; NIV, noninvasive ventilation; SD, standard deviation.

The blood test analyses revealed a slight increase in D-dimer values 2–3 months after the onset of COVID-19. Other proinflammatory and coagulation parameters showed values within the normal range (Table 2).

**Table 2:** Blood test analyses at follow-up.

Parameters	Median (IQR)	Reference values
D-dimer (ng/ml)	510 (280, 780)	<500
PT/INR (s)	1.0 (1.0, 1.1)	0.8–1.1
C-reactive protein (mg/dl)	0.2 (0.1, 0.4)	<1.0
tPA (units/ml**)**	1.0 (0.5)	<1.1

Blood test analyses of 47 patients with severe COVID-19 pneumonia 2–3 months following discharge. IQR, interquartile range; PT/INR, prothrombin time (International Normalized Ratio); tPA, tissue plasminogen activator.

Lung ultrasound images revealed normal pattern in 28 patients (51.9%) as represented by A-lines or <3 separated B-lines, whereas 21 patients (38.9%) showed large consolidations in the images, and 5 (9.3%) presented mild morphological alterations (Table 3). Four patients (out of 58 in the total sample) were not assessed by lung ultrasound because CT scan was used instead.

**Table 3:** Lung ultrasound findings.

LUS findings	*n* (%)
Score 0. A-lines or <3 separated B-lines	28 (51.9)
Score 1. At least three B-lines or coalescent B-lines occupying <50% of the screen with a clearly irregular pleural line	4 (7.4)
Score 2. Coalescent B-lines occupying >50% of the screen with/without a clearly irregular pleural line	1 (1.9)
Score 3. Large consolidations	21 (38.9)

Lung ultrasound findings of 54 patients with severe COVID-19 pneumonia at 2–3 months following discharge.

Diaphragm exams performed by ultrasound showed excursions ranging from 0.5 cm–3.8 cm in quiet breathing, 1.6 cm–7.6 cm in deep breathing, and 1.3 cm–3.8 cm in voluntary sniff, whereas thickness ranged from 1.1 to 3.0 mm. Patients with normal and abnormal lung ultrasound findings presented statistical difference in the diaphragmatic excursion in deep breathing (*p *= 0.029). No difference was observed in terms of thickness. The mean values and standard deviations are shown in Table 4.

**Table 4:** Diaphragm ultrasound exam.

Parameters	Total participants, Mean (SD)	Normal LUS, Mean (SD)	Abnormal LUS, Mean (SD)	*p* value	Cohen's d
Excursion in quiet breathing (cm)	1.9 (0.5)	2.0 (0.4)	1.8 (0.7)	0.14	0.41
Excursion in deep breathing (cm)	5.1 (1.4)	5.6 (1.1)	4.7 (1.5)	0.029^a^	0.63
Excursion in voluntary sniff (cm)	2.2 (0.6)	2.2 (0.6)	2.2 (0.6)	0.66	0.12
Thickness (mm)	2.1 (0.5)	2.1 (0.5)	2.0 (0.5)	0.83	0.05

Diaphragm ultrasound exam in Total participants (*n* = 55), participants with normal LUS findings (*n* = 27), and abnormal LUS findings (*n* = 28). LUS, lung ultrasound. Cohen's d values for effect size: <0.2 no effect, 0.2 to 0.5 small, >0.5 to 0.8 medium, and >0.8 large.

^a^ Statistically significant.

Lung function tests results are presented in Table 5. We observed FEV_1_ <80% in 15 (25.8%) patients, out of those 11 (73.3%) showed concomitant FEV_1_/FVC ≥70%. Overall, restrictive pattern in spirometry (FEV_1_ <80% and FEV_1_/FVC ≥70%) was observed in 18.9% of patients in our sample. Eighteen patients (58.0%) had abnormal DLCO, out of those 8 (25.8%) had a mild reduction (DLCO 61%–75%), 9 (29.0%) a moderate reduction (DLCO 41%–60%) and 1 (3.2%) a severe reduction (DLCO <40%).

**Table 5:** Lung function parameters (spirometry and DLCO) of 57 patients.

Parameters	Total participants, Mean (SD)	Normal LUS, Mean (SD)	Abnormal LUS, Mean (SD)	*p* value	Cohen's d
FVC (L)	3.3 (1.0)	3.7 (0.9)	3.0 (0.9)	<0.01^a^	0.83
FEV_1_ (L)	2.7 (0.8)	3.0 (0.8)	2.4 (0.7)	<0.01^a^	0.75
FVC% predicted	85.7 (19.0)	95.0 (14.8)	77.4 (18.7)	<0.01^a^	1.04
FEV_1_% predicted	94.0 (21.9)	103.4 (19.6)	85.5 (20.7)	<0.01^a^	0.89
FEV_1_/FVC% predicted	80.1 (6.0)	79.2 (6.7)	80.8 (6.5)	0.36	0.24
DLCO% predicted	68.9 (18.8)	74.6 (11.9)	63.0 (21.2)	0.17	0.49
KCO% predicted	87.2 (15.6)	88.3 (15.8)	86.7 (15.8)	0.79	0.10

Lung function parameters in Total participants (*n* = 55), participants with normal LUS findings (*n* = 27), and abnormal LUS findings (*n* = 28). DLCO, diffusing capacity of the lungs for carbon monoxide; FEV_1_, forced expiratory volume in one second; FVC, forced vital capacity; KCO, carbon monoxide transfer coefficient; LUS, lung ultrasound. Cohen's d values for effect size: <0.2 no effect, 0.2 to 0.5 small, >0.5 to 0.8 medium, and >0.8 large.

^a^ Statistically significant.

**Figure 1:**
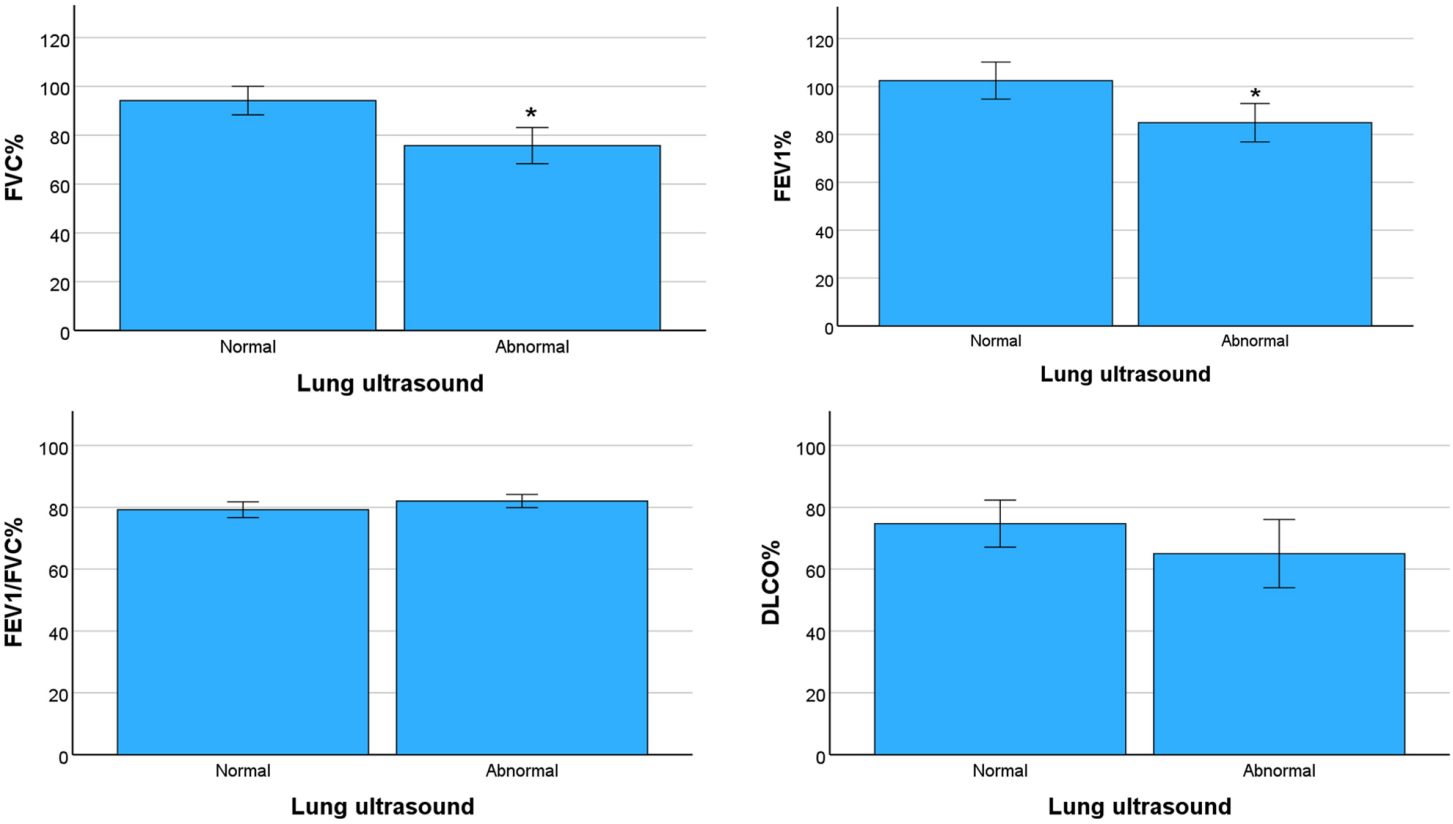
Comparison of lung ultrasound scores grouped in normal (score 0) or abnormal (scores 1, 2 and 3) with parameters of lung function. FVC%: percent predicted forced vital capacity, FEV_1_%: percent predicted forced expiratory volume in the first second, FEV_1_/FVC%: percent predicted FEV_1_/FVC ratio, DLCO%: percent predicted diffusing capacity of the lungs for carbon monoxide. Data represent mean values and bars the standard error of the mean. *p < 0.01.

Patients with abnormal LUS findings presented significant statistical differences in FVC% and FEV_1_% (*p *< 0.01) when compared with patients presenting normal LUS findings. No significant difference was observed in the other parameters of lung function (Figure 1).

Variables related to exercise capacity, physical activity levels (IPAQ), and health-related quality of life (K-BILD) are reported in Table 6. The average distance in the 6MWT was 439.9 meters (64.7% predicted), with 32 patients (55.1%) presenting desaturation larger than 3%. The average number of repetitions in the sit-to-stand test was 13.6. Twenty-eight (48.3%) participants self-reported moderate level of physical activity, 16 (27.6%) self-reported intense levels, and 14 (24.1%) low levels of physical activity. The mean total score in the K-BILD questionnaire of quality of life was 79.5 points.

**Table 6:** Exercise capacity, physical activity levels (IPAQ), and health-related quality of life (K-BILD) of 58 patients.

Variable	Mean (SD)
6MWT distance (meters), mean (SD)	439.9 (87.1)
6MWT distance (% predicted), mean (SD)	64.7 (16.8)
6MWT lowest SpO_2_ (%), mean (SD)	94.5 (3.1)
6MWT desaturation (%), mean (SD)	3.4 (2.8)
6MWT HR baseline (bpm), mean (SD)	77.2 (2.9)
6MWT HR highest (bpm), mean (SD)	107.7 (16.3)
30 s-STS test (number of repetitions), mean (SD)	13.6 (5.5)
IPAQ intense category, *n* (%)	16 (27.6)
IPAQ moderate category, *n* (%)	28 (48.3)
IPAQ low category, *n* (%)	14 (24.1)
K-BILD (total score), mean (SD)	79.5 (16.2)

6MWT, six-minute walk test; 30 s-STS, 30 s sit-to-stand; bpm, beats per minute; HR, heart rate; IPAQ, international physical activity questionnaire; K-BILD, king interstitial lung disease questionnaire; SpO_2_, oxygen saturation.

## 4. Discussion

This study followed up patients with severe respiratory failure due to COVID-19 during the first wave of the pandemic at 2–3 months after hospital discharge. Although a significant part of them presented with normal overall functional status, they displayed abnormalities in LUS, pulmonary function, and diaphragmatic excursion.

The acute phase of COVID-19 is characterized by an inflammatory cascade that may induce venous thromboembolic complications (32, 33). Our results show values of prothrombin time, C-reactive protein and tPA within the normal range values, and a trend to normalization of D-dimer after 2–3 months from hospital discharge. Similarly (33), found a slight and persistent alteration of D-dimer, fibrinogen, and IL-6 in a non-negligible percentage of COVID-19 patients (32.2%, 61%, and 32.2%, respectively) 3–6 months after discharge. Although the clinical relevance of these data is not clear, the authors suggest the risk for thromboembolic complications quickly decreases during the convalescence phase.

It is well known that prolonged IMV poses a threat in developing secondary infections and mobility restrictions that may lead to long-term complications (34). In the present study, we found lung ultrasound abnormalities in nearly half (48.1%) of the individuals 2–3 months after hospital discharge. This finding is supported by a similar study carried out in the first Italian epidemic wave showed that 59% of patients had an interstitial pulmonary syndrome observed in LUS 2 months from acute phase (35). Considering all participants in the present study either required invasive or noninvasive mechanical ventilation in their hospital stay, we could attribute the LUS abnormal findings to the degree of COVID-19 severity.

The pulmonary function tests of this study displayed 18.9% of patients with impaired spirometry values, considering FEV1 <80% and FEV1/FVC ≥70% (36). Other studies show a similar proportion (17%) of spirometry alterations among COVID-19 survivors (37, 38). A recent study described that critical patients had greater improvements in pulmonary function tests during the first year after COVID-19, when compared to moderate and severe patients (39).

Diaphragmatic excursion is 1 cm–2 cm during tidal breathing and 7 cm–11 cm during deep inspiration in healthy subjects (40). On the other hand, diaphragm dysfunction is observed in 10% of the patients with COVID-19 in the critical unit and it has been reported that the diaphragm thickness declines during the hospital stay (9, 41, 42). Despite the long-term implications are still unclear, the participants of our study presented significant less excursion in deep breathing consistent with the lung morphological alterations observed in LUS exam. Although no difference was observed in the diaphragm thickness of patients with or without LUS alterations, an apparent normal diaphragm at some point could become exhausted because of less compliant lungs. Therefore, extrapulmonary damage caused by COVID-19 to the diaphragm cannot be ruled out.

Both the 6MWT and the 30-s STS test complement each other as they evaluate different aspects of physical capacity: the 6MWT relates to endurance and cardiovascular fitness, whereas the 30-s STS refers to lower extremity muscle strength and power (26, 28). The participants of our study presented with decreased cardiovascular fitness as they walked an average of 64.7% predicted distance and more than half of them desaturated during the 6MWT. These data are consistent with an Italian observational study which attributed that patients with more severe COVID-19 pneumonia (PaO_2_/FiO_2_ nadir ≤200) had a lower minimum SpO_2_ and a higher desaturation % in the 6MWT at 2- and 6-month follow-up (35). The functional status of COVID-19 survivors at the critical unit and hospital discharge has been reported in a few studies. Worse functional status at hospital discharge was associated with longer IMV duration, older age, higher number of comorbidities, hypertension, diabetes, chronic obstructive pulmonary disease, and immunosuppression (43). A study by (44) found that 80% of COVID-19 recovered cases have diverse degrees of functional restrictions ranging from negligible (63.1%), slight (14.4%), moderate (2%), to severe (0.5%) based on “Post-COVID-19 Functional Status” (PCFS). It has also been shown that a large proportion of patients had worsened quality of life, reduced functional status (assessed by PCFS), and persistent symptoms compared with their pre-COVID-19 status at 6 months after ICU admission (45). We chose to assess our patients with the 30 s-STS test because it allowed optimization of resources in the pandemics context as it requires low time of execution, equipment and space (28). The 30 s-STS power was found to indicate global muscle wasting in COVID-19 survivors one month after hospitalization (28). Conversely, the patients in our study presented average values of 30 s-STS within the normal range for the healthy population, thus suggesting a recovery of lower extremity strength and power at 2–3 months post discharge.

Nearly half (48.3%) of the participants of our study perceived their levels of physical activity as moderate, following by 27.6% who self-reported intense levels, and 24.1% low levels of physical activity, along with normal health-related quality of life. It particularly came to our attention that many participants of our sample were very scared of their prognosis after hospital discharge in the first wave of the pandemic, and likewise grateful for the effort made by the frontline healthcare professionals. Taken together, we believe the lack of significant alterations in their self-reported levels of physical activity and quality of life were due to a positive attitude and self-management towards their own recovery. Contrary to our results, data reported from an Italian cohort of severe COVID-19 patients show deterioration on the physical component of quality of life one year after ICU discharge, attributing the female gender to influence negatively the physical function (20). Considering the high proportion of males (67.2%) enrolled in the present study, the overall normal health-related quality of life self-reported by the patients could have been dampened.

A plethora of evidence sustain that critical COVID-19 patients have prominent long-term deterioration of exercise capacity and physical status (39, 43–45). However, it has been lately noticeable that clinical manifestations of COVID-19 are heterogeneous, and the disease severity does not necessarily impact on exercise capacity of survivors. Some authors attribute this finding to different degrees of adaptation to inflammatory responses affecting cardio-respiratory efficiency (2, 46).

The biggest strength of this study is the comprehensive assessment of patients with severe COVID-19 pneumonia after hospital discharge. Considering the large heterogeneity of information on the subacute clinical and functional manifestations caused by COVID-19, this study reports that impaired pulmonary and diaphragmatic function at 2–3 months post discharge could be related to the persistence of LUS abnormalities in this time window. However, this study presents limitations regarding the limited sample size (single center), therefore multicenter studies are necessary to extrapolate the results to other regions. In this line, low external validity could be considered another limitation because of the convenience sampling employed in this study. Additionally, the data extracted from the medical record was also limited by the retrospective nature of this study. Lastly, no lung function, exercise capacity, or ultrasound results were gathered before COVID-19 pandemic, therefore the lack of baseline data hinders a longitudinal analysis. More extended follow-up is necessary to better understand the possible long-term events caused by COVID-19.

## 5. Conclusions

This study provides evidence that patients with severe COVID-19 pneumonia who present abnormalities in LUS imaging concomitantly display significant impaired lung function, and lesser mobility of the diaphragm 2–3 months post discharge. Our findings reaffirm the need of assessing the functional status of the survivors to monitor the evolution of pulmonary and systemic damage, and target specific rehabilitation interventions to tackle COVID-19 functional sequelae.

## Data Availability

Research data cannot be shared publicly due to confidential ethical reasons according to the Research Ethics Committee of Granollers General Hospital (Spain). Anonymous datasets are available upon reasonable request to the corresponding author.
